# Interplay between Nrf2 and αB-crystallin in the lens and heart of zebrafish under proteostatic stress

**DOI:** 10.3389/fmolb.2023.1185704

**Published:** 2023-07-28

**Authors:** Jinhee Park, Samantha MacGavin, Laurie Niederbrach, Hassane S. Mchaourab

**Affiliations:** From the Department of Molecular Physiology and Biophysics, Vanderbilt University, Nashville, TN, United States

**Keywords:** Nrf2, oxidative stress, chaperones, small heat shock proteins, αB-crystallin

## Abstract

A coordinated oxidative stress response, partly triggered by the transcription factor Nrf2, protects cells from the continual production of reactive oxygen species. Left unbuffered, reactive oxygen species can lead to protein aggregation that has been implicated in a spectrum of diseases such as cataract of the ocular lens and myopathy of the heart. While proteostasis is maintained by diverse families of heat shock proteins, the interplay between the oxidative and proteostatic stress responses in the lens and heart has not been investigated. Capitalizing on multiple zebrafish lines that have compromised function of Nrf2 and/or the two zebrafish small heat shock proteins αBa- and αBb-crystallin, we uncovered a transcriptional relationship that leads to a substantial increase in αBb-crystallin transcripts in the heart in response to compromised function of Nrf2. In the lens, the concomitant loss of function of Nrf2 and αBa-crystallin leads to upregulation of the cholesterol biosynthesis pathway, thus mitigating the phenotypic consequences of the αBa-crystallin knockout. By contrast, abrogation of Nrf2 function accentuates the penetrance of a heart edema phenotype characteristic of embryos of αB-crystallin knockout lines. Multiple molecular pathways, such as genes involved in extracellular interactions and implicated in cardiomyopathy, are revealed from transcriptome profiling, thus identifying novel targets for further investigation. Together, our transcriptome/phenotypic analysis establishes an intersection between oxidative stress and chaperone responses in the lens and heart.

## Introduction

Oxidative stress presents a sustained challenge to long-lived cells, such as the lens fiber cells, and cells with high metabolic requirements, such as the cardiac myocytes (Truscott, 2005; Bartz et al., 2015). Unbuffered reactive oxygen species (ROS) are associated with multiple deleterious effects on cellular homeostasis, which includes DNA damage, protein oxidation, and organelle dysfunction (Auten and Davis, 2009). A central player in the maintenance of cellular oxidative balance is the transcription factor Nrf2 (Tonelli et al., 2018). Nrf2 regulates antioxidant responses by turning on the expression of enzymes that eliminate ROS and maintains the redox balance (Tonelli et al., 2018). Through modulation of its interaction with Kelch-like ECH-associated protein 1 (Keap1), oxidative stress drives Nrf2 translocation into the nucleus to activate genes involved in glutathione synthesis, detoxification, elimination of ROS, and drug excretion (Yamamoto et al., 2018).

In long-lived cells, protein damage that leads to the loss of stability and/or solubility can induce the formation of protein aggregates. A prominent example of the deleterious effects of protein aggregation occurs in the ocular lens. Accumulation of age-dependent damage to lens proteins induced by multiple factors, such as UV radiation and oxidation, leads to changes in their stability and solubility (Kopylova et al., 2011; Vetter et al., 2020; Luo et al., 2021). Gradual nucleation of aggregation-prone proteins eventually leads to large condensates that result in lens opacification, light scattering, and potentially age-related cataract (Moreau and King, 2012). Age-related cataract is one of the world’s most common causes of blindness (Murthy et al., 2001; Duerksen et al., 2003; Dunzhu et al., 2003; Thulasiraj et al., 2003; Zhao et al., 2010; Zheng et al., 2011). Numerous lines of evidence suggest that oxidative stress is a leading risk factor for cataract formation (Wu and Leske, 2000; Truscott, 2005; Shui et al., 2006; Beebe et al., 2010). Remarkably, lens protein’s relatively rich cysteine and methionine content makes it highly prone to oxidation by reactive oxygen species (ROS) (Truscott and Augusteyn, 1977; Garner and Spector, 1980a; Garner and Spector, 1980b). Over 90% of cysteine residues and 50% of methionine residues are oxidized in cataract patients’ lenses (Truscott and Friedrich, 2019). Balancing the life-long danger of oxidative stress, the lens possesses a robust antioxidant defense system to scavenge and detoxify ROS. Glutathione (GSH) is present at exceedingly high concentrations in the lens, allowing it to resist oxidative damage (Giblin, 2000). The implied mechanistic link between Nrf2 activity and cataract formation has sparked interest in Nrf2 as a potential therapeutic target for cataract treatment and prevention (Liu et al., 2017; Rowan et al., 2021; von Otter et al., 2010).

The programmed elimination of light-scattering organelles in lens fiber cells is essential to achieve the lens’s optical properties (Bassnett, 2009). As a result, proteome turnover is exceedingly low, and lens proteins must remain for a lifetime. Proteostasis in the quiescent vertebrate lens is maintained almost exclusively by chaperone activity of the resident small heat shock proteins (sHSPs), αA- and αB-crystallin, which have been hypothesized to play a central role in preventing the aggregation of unstable and damaged proteins (Horwitz, 1992; Kumar and Reddy, 2009; Slingsby et al., 2013; Kaiser et al., 2019). In addition, mutations in lens α-crystallins have been associated with congenital cataracts (Sun et al., 2017; Berry et al., 2020). Thus, through their complementary roles in preventing protein damage and inhibiting protein aggregation, Nrf2 and sHSPs are important factors in the maintenance of the lens’s optical properties.

Unlike αA-crystallin, which is mainly expressed in the ocular lens, αB-crystallin is detected in multiple tissues, such as in the heart, brain, skeletal muscles, kidneys, and extracellular matrix (Bhat and Nagineni, 1989). Transcriptional regulation of *αB-crystallin* in mammals is tissue specific and triggered by heat shock and other stress stimuli, which include arsenite/cadmium, hypertonic/osmotic stresses, and oxidative stress, and is regulated by stress-activated proteins such as heat shock factor 1 (hsf1) or transcription factor AP1 (Klemenz et al., 1991). αB-crystallin has been implicated in maintaining cardiac homeostasis through a spectrum of roles in myocytes. It interacts with cytoskeletal proteins such as Titin, which serves as a molecular spring for passive muscle elasticity (Swist et al., 2020). It has indeed been reported that high cardiomyocyte stiffness, which is highly correlated with aortic stenosis and dilated cardiomyopathy, was corrected by αB-crystallin through suppression of Titin aggregation (Franssen et al., 2017). In addition, αB-crystallin in myocytes has a protective role in response to ischemia-reperfusion stress, a condition that elevates reactive oxygen species (ROS) production (Chis et al., 2012). Finally, the R120G mutation in the *αB-crystallin* gene is associated with congenital cataracts and muscular diseases, such as cardiomyopathy (Bova et al., 1999; Boelens, 2014). R120G-related cardiomyopathy is characterized by reductive stress, desmin aggregation in inclusion bodies, and ventricular dysfunction (Kannan et al., 2013). Collectively, these studies suggest that αB-crystallin plays a pleiotropic role in various cellular activities such as stabilization of the cytoskeletal structure, protein quality control, cell differentiation, and apoptosis. Thus, similar to lens fiber cells, sHSPs and Nrf2 are involved in the oxidative and proteostasis response in cardiomyocytes.

While multiple lines of evidence suggest a convergence between the roles of sHSPs, particularly αB-crystallin, and the oxidative stress response (Shin et al., 2009; Chis et al., 2012; Christopher et al., 2014; Kim et al., 2020), there has been no systematic investigation of the direct link between them nor has the *in vivo* molecular mechanism underlying this link been explored. In this study, we investigated the mechanistic relationship between tissue-specific regulation of zebrafish *αBa-* and *αBb-crystallin* and oxidative stress due to Nrf2 depletion, focusing on the lens and heart tissues where αB-crystallin’s physiological roles have been demonstrated unequivocally. In zebrafish, two *αB-crystallin* paralogs have been identified due to gene duplication (Smith et al., 2006), αBa-crystallin and αBb-crystallin*.* Zebrafish *αBa-crystallin* transcripts are predominantly expressed in the lens, whereas *αBb-crystallin* is more widely expressed in multiple tissues, such as in the lens, muscle, and brain (Smith et al., 2006). Importantly, recombinant zebrafish αBa-crystallin exhibits more potent chaperone-like activity than αBb-crystallin *in vitro* (Smith et al., 2006; Koteiche et al., 2015). These findings suggest that the two zebrafish αB-crystallins are subjected to divergent selection pressures to meet the challenges of distinct physiological functions.

Taking advantage of several zebrafish lines that have compromised αB-crystallins and/or Nrf2 function, we uncovered a transcriptional coupling between *Nrf2* and *αB-crystallins* genes. This coupling manifests in the modulation of the lens and heart phenotypes of αB-crystallins’ loss-of-function zebrafish lines. Unexpectedly, Nrf2 deficiency, which increases the oxidative load, suppressed the lens defect phenotype in the *αBa-crystallin* knockout zebrafish embryos but enhanced the heart edema phenotype characteristic of these lines. Transcriptome analysis identified distinct molecular pathways activated in response to impaired *Nrf2* function in *αBa-crystallin*–depleted lens and heart tissues. We found that Nrf2 deficiency activates cholesterol biosynthesis pathways in the *αBa-crystallin* mutated lens. By contrast, Nrf2 deficiency drives transcriptional change in the extracellular region and tight junction pathways in the heart tissue of *αBa-crystallin* mutant. To our knowledge, this is the first evidence of the role of sHSPs in the oxidative stress response and sets the stage for an in-depth investigation of how transcriptional link to Nrf2 is mediated.

## Results

### Tissue-specific Nrf2 regulation of αB-crystallin transcripts

To investigate how oxidative stress pathways modulate αB-crystallin expression and/or function, we utilized sensitized zebrafish lines bearing a mutation within *nrf2* that substantially reduces its activity (Mukaigasa et al., 2012). The *nrf2* ^*fh318*^ zebrafish line has a mutation in the basic region of the DNA-binding domain (exon 5), which leads to decreased transcriptional induction of Nrf2 target genes, which include antioxidant enzymes. We observed that the mRNA level of *nrf2* downstream genes such as *gstp1* and *prdx1* were lower in the lens and heart tissues of *nrf2* ^fh318/fh318^ lines relative to WT fish (Supplementary Figure S1). These results are consistent with previous studies, although ours were carried out in the absence of oxidative stress (Mukaigasa et al., 2012). We also confirmed that the level of *αBa-crystallin* and *αBb-crystallin* transcripts was relatively higher in the lens than was in the heart and brain tissues, whereas the levels of *nrf2* transcripts in the heart and brain were higher than that in the lens (Supplementary Figure S2).

**FIGURE 1:**
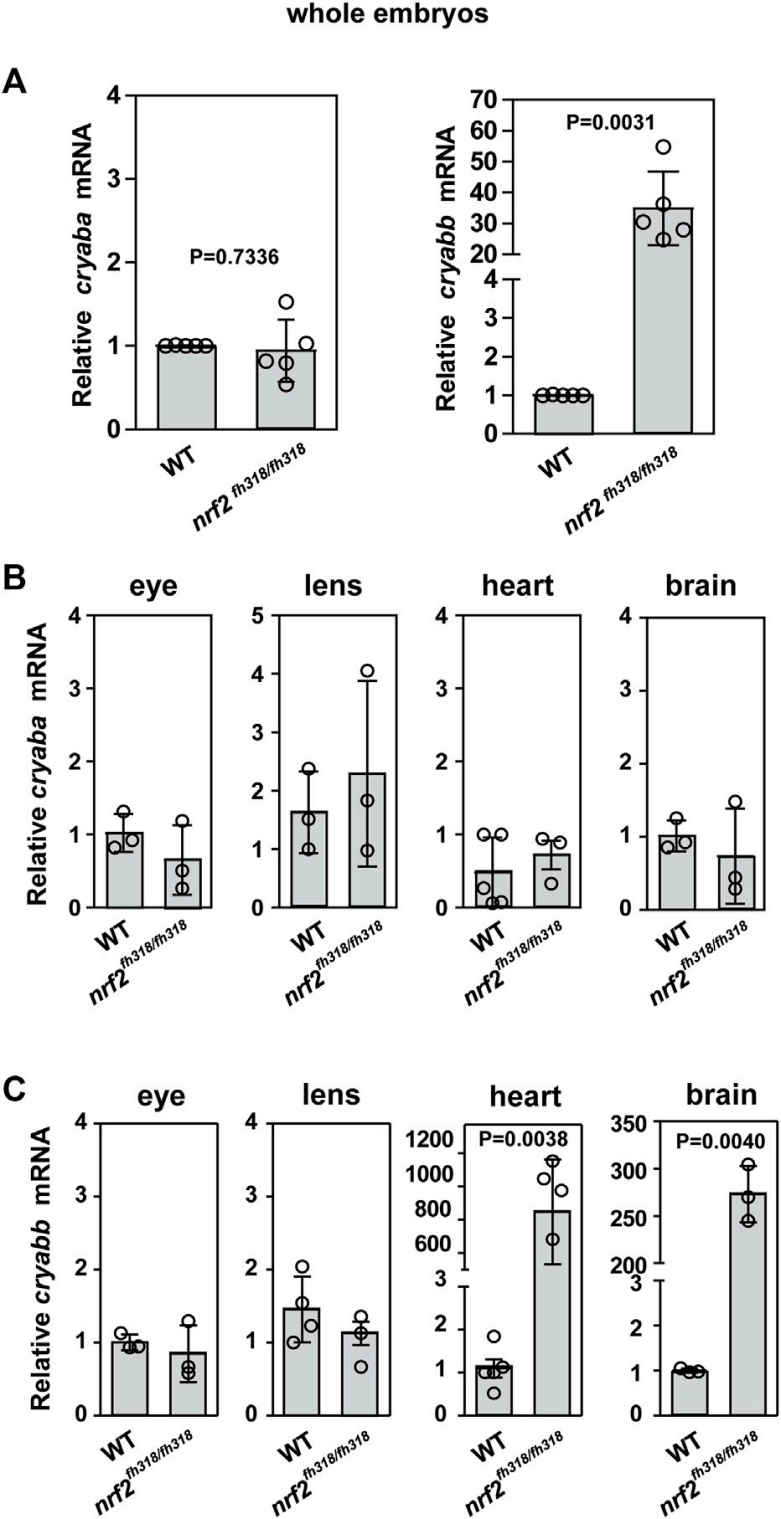
Zebrafish *cryab* transcripts are regulated in a tissue-dependent manner. **(A)** Relative expression of *cryaba* and *cryabb* genes was compared between WT and *nrf2* ^fh318/fh318^ embryos at 4 dpf using qRT-PCR analysis. Data are expressed as mean ± SD from five independent measurements. The relative mRNA levels of *cryaba* **(B)** and *cryabb* **(C)** were measured between WT and *nrf2* ^fh318/fh318^ by qRT-PCR in the eyes, lens, heart, and brain tissues of 10-month-old zebrafish. Data are expressed as mean ± SD from at least *n* = 3 for brain, eye, lens, and heart. *p*-values were calculated using two-tailed *t*-test.

To test if αB-crystallin expression is affected by Nrf2 deficiency, mRNA levels of *αBa-crystallin* and *αBb-crystallin* genes (hereafter, we use the nomenclature “*cryaba*” and “*cryabb*” for simplicity) were compared between WT and *nrf2* ^fh318/fh318^ embryos by the qRT-PCR analysis. Although the levels of *cryaba* transcript were not changed between WT and *nrf2* ^fh318/fh318^, *cryabb* transcription was highly upregulated in Nrf2-deficient embryos (Figure 1A). This modulation of *cryabb* transcripts appears tissue specific. *cryaba* transcripts were not substantially changed in the whole eyes, lens, heart, and brain tissues of *nrf2*-mutated zebrafish relative to WT (Figure 1B). By contrast, *cryabb* transcripts were upregulated strongly in the heart and brain tissues of Nrf2-deficient zebrafish (Figure 1C; Supplementary Figure S3). To test whether transcription of *cryabb* can be upregulated in response to increased oxidative stress, we measured the level of *cryabb* mRNA after treating WT embryos with 800 μM tert-Butyl hydroperoxide (tBHP) for 2 h at 4 days post fertilization (dpf). We observed an increase in the *cryabb* transcript (∼1.5 fold) with tBHP treatment (Supplementary Figure S4B), although to a lesser extent when compared to the reduced activity of Nrf2.

To investigate crosstalk between αB-crystallin and *nrf2*, we assessed the regulation of *nrf2* and *nrf2* downstream targets, such as antioxidant genes, in αB-crystallin knockout lines (*cryaba* ^−/−^, *cryabb* ^−/−^, *cryaba* ^−/−^, and *cryabb* ^−/−^ double mutants). Although the level of *nrf2* mRNA was not significantly changed in WT and αB-crystallin knockout lines, its targets, *gpx1a* and *prdx1*, were upregulated in *cryabb* ^*−/−*^ embryos (Supplementary Figure S5). Taken together, these results suggested a transcriptional link between *nrf2* and *cryabb* but not *cryaba*. Further detailed experiments are required to examine the tissue-specific mechanisms underlying the upregulated antioxidant genes in the *cryabb* ^−/−^ lines.

### Nrf2 deficiency suppresses lens defects in the *cryaba* ^*−/−*^ mutant

**FIGURE 2:**
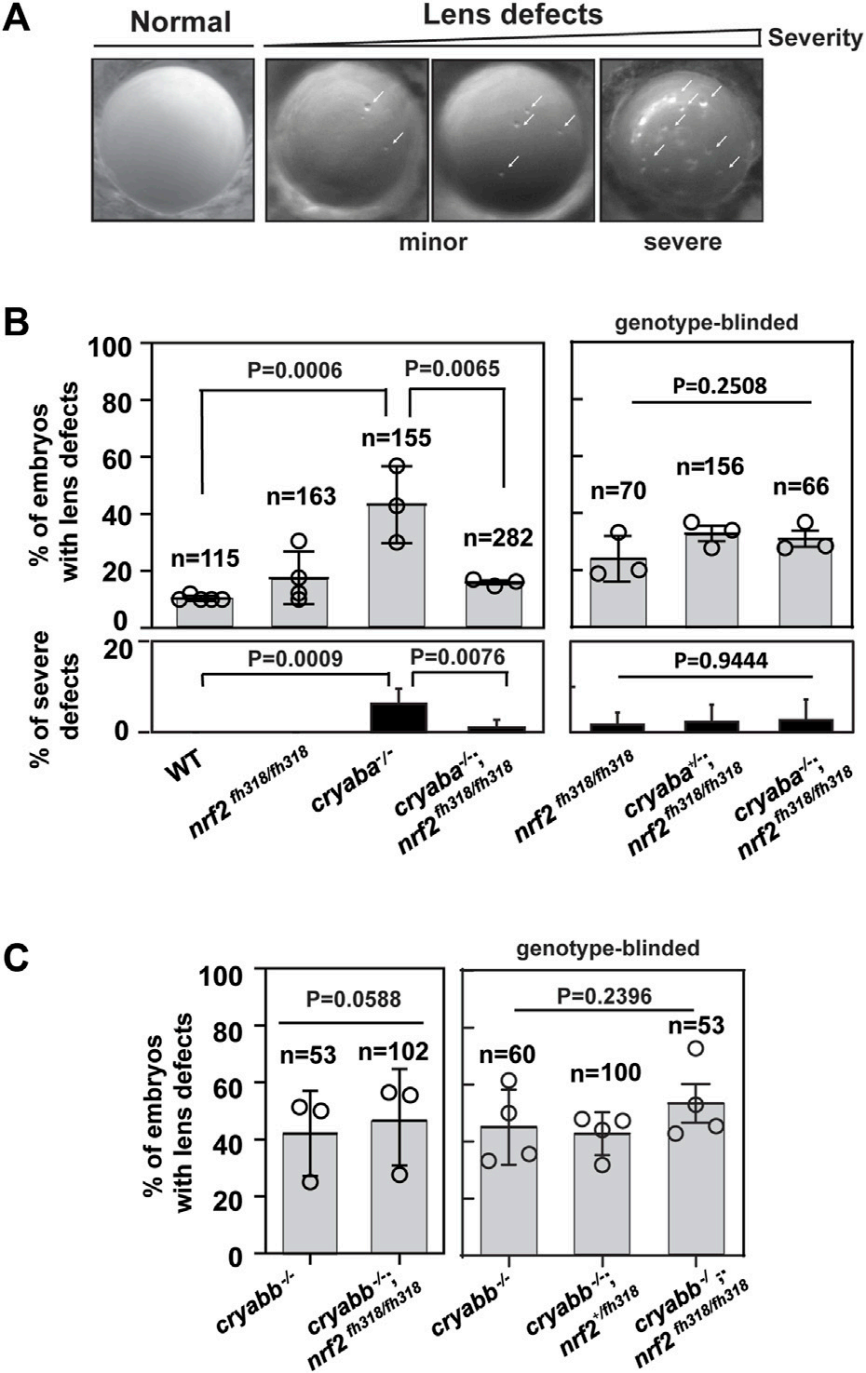
Nrf2 deficiency rescues lens defects in *cryaba* ^*−/−*^ but not in *cryabb* ^*−/−*^. **(A)** Representative images of lens defects in zebrafish embryos at 4 day post fertilization (dpf). [**(B)**, left panel] Percentage of embryos showing lens defects for WT, *nrf2* ^fh318/fh318^, *cryaba* ^−/−^, and *cryaba* ^−/−^; *nrf2* ^fh318/fh318^ was compared. [**(B)**, right panel] To confirm the previous results, embryos from *cryaba* ^+/−^; *nrf2* ^fh318/fh318^ incross were collected, and the lens defect of each embryo was measured in a genotype-blinded at 4 dpf. Then, *cryaba* genotyping of individual embryos was determined to compare the percentage of lens defects between *nrf2* ^fh318/fh318^ and *cryaba* ^−/−^; *nrf2* ^fh318/fh318^. [**(C)**, left panel] Percentage of lens defect between *cryabb* ^−/−^ and *cryabb* ^−/−^; *nrf2* ^fh318/fh318^ embryos. [**(C)**, right panel] Embryos from *cryabb* ^−/−^; *nrf2* ^fh318/+^ incross were collected, and lens abnormalities were examined before *nrf2* genotyping. Then, the percentage of lens defects between *cryabb* ^−/−^ and *cryabb* ^−/−^; *nrf2* ^fh318/fh318^ was analyzed. Data are expressed as mean ± SD from at least three independent experiments. n numbers indicate the total number of embryos across the independent experiments. *p*-values were calculated using one-way ANOVA and two-tailed *t*-test.

In light of the aforementioned transcriptional relationship, we explored its consequences on the phenotypic manifestation of the loss of αB-crystallin function. Previously, we showed that knockout of either αB-crystallin causes lens defects in zebrafish embryos (Mishra et al., 2018; Zou et al., 2015). αB-crystallin mutant embryos, *cryaba* ^−/−^ and *cryabb* ^−/−^, presented lens abnormalities at 4 dpf (Figure 2A). The phenotypic features characterizing these lines appeared as round puncta spread across the lens. These puncta lead to opacity and changes in light scattering. Consistent with our previous results, ∼50% and ∼30% of *cryaba* ^−/−^ and *cryabb* ^−/−^embryos exhibited lens abnormalities, respectively (Supplementary Figure S6). In comparison, *nrf2* ^*fh318/fh318*^ showed a slightly increased percentage of lens defects (∼20%) relative to WT embryos (∼10%) (Figure 2B).

**FIGURE 3:**
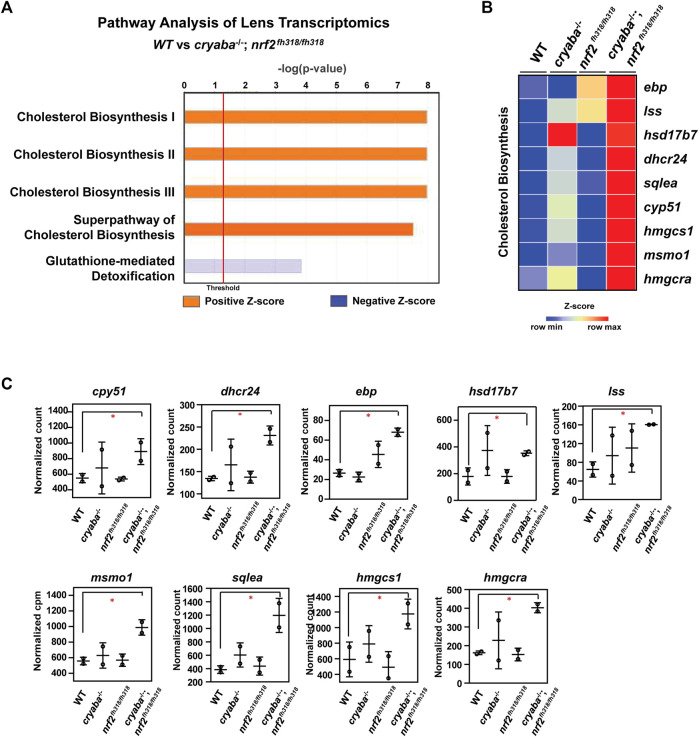
Cholesterol biosynthesis pathway is elevated in the lens of *cryaba* ^−/−^; *nrf2* ^fh318/fh318^ **(A)** Lens RNA-seq data were analyzed through ingenuity pathway analysis (www.ingenuity.com). Orange bars that cross the threshold line (*p* < 0.05) indicate upregulated pathways in the lens of *cryaba* ^−/−^; *nrf2* ^fh318/fh318^ when compared to WT. **(B)** Heatmap of enriched genes in the superpathway of cholesterol biosynthesis. Z-scores were calculated for each gene, and these were plotted instead of the normalized expression values. **(C)** Bar charts represent the normalized count of each transcript in the cholesterol biosynthesis pathway in the lens tissues of WT, *nrf2* ^fh318/fh318^, *cryaba* ^−/−,^ and *cryaba* ^−/−^; *nrf2* ^*fh318/fh318*^. *Indicates false discovery rate (FDR) < 0.05.

To investigate how Nrf2 deficiency affects lens development in the absence of αB-crystallin, we generated αB-crystallin/Nrf2 double knockout lines (*cryaba* ^+/−^; *nrf2* ^fh318/fh318^, *cryaba* ^−/−^; *nrf2* ^fh318/fh318^, *cryabb* ^−/−^; *nrf2* ^fh318/+^, and *cryabb* ^−/−^; *nrf2* ^fh318/fh318^). Then, we compared the penetrance of lens abnormalities in the progeny of WT, *nrf2* ^fh318/fh318^, *cryaba* ^−/−^ and *cryaba* ^−/−^; *nrf2* ^fh318/fh318^ embryos. Strikingly, we found that the elevated level of lens defects in the *cryaba* ^−/−^ lines was suppressed in the *cryaba* ^*−/−*^/*nrf2* ^fh318/fh318^ (Figure 3B, left panel). To confirm this result, we in-crossed *cryaba* ^+/−^; *nrf2* ^fh318/fh318^ adult zebrafish and screened their embryos for lens defects in a genotype-blinded experimental protocol. After the lenses were imaged and sorted into normal and defective lens groups (Figure 2A), we categorized the *cryaba* genotype of individual embryos into three groups (*cryaba* ^+/+^; *nrf2* ^fh318/fh318^, *cryaba* ^+/−^; *nrf2* ^fh318/fh318^, and *cryaba* ^−/−^; *nrf2* ^fh318/fh318^). The percentage of lens defects of *cryaba* ^−/−^; *nrf2* ^fh318/fh318^ embryos is similar to the level of *nrf2* ^fh318/fh318^ (Figure 3B, right panel). The results are consistent with the conclusion that Nrf2 deficiency reduces the penetrance of the lens phenotype induced by αBa-crystallin loss of function. Moreover, among embryos with lens defects, the percentage of severe lens defects of *cryaba* ^*−/−*^-mutated embryos was higher than that of *cryaba* ^−/−^; *nrf2* ^fh318/fh318^, indicating that *nrf2* deficiency alleviates the severity of lens defects (Figure 3B, bottom panel). By contrast, we did not observe large changes in the percentage of lens defects between *cryabb* ^−/−^ and *cryabb* ^−/−^; *nrf2* ^fh318/fh318^ embryos, suggesting that the lens defects in *cryabb* KO lines cannot be rescued by abrogation of Nrf2 function (Figure 2C).

### Upregulation of cholesterol biosynthesis pathway in the lens of *cryaba* ^−/−^; *nrf2* ^fh318/fh318^ double mutant lines

To identify the molecular pathway(s) mediating the suppression of lens defects in the *cryaba* mutant by Nrf2 deficiency, we performed a high-throughput transcriptome profiling on adult zebrafish lens tissues from the WT, *cryaba* ^−/−^, *nrf2* ^fh318/fh318^, and *cryaba* ^−/−^; *nrf2* ^fh318/fh318^ lines. Differentially expressed (DE) genes between the groups were determined using Illumina’s DRAGEN pipeline. Then, the DE genes were processed using the Ingenuity Pathway Analysis (IPA) to define the significantly regulated signaling pathways (Kramer et al., 2014). Interestingly, cholesterol biosynthesis was identified as the primary upregulated pathway in the lens of *cryaba* ^−/−^; *nrf2* ^fh318/fh318^ when compared to that of WT or *cryaba* ^−/−^ (Figure 3A; Supplementary Figure S7B). mRNA of the gene-encoding enzymes involved in the superpathway of cholesterol biosynthesis—*cyp51*, *dhcr24*, *ebp*, *lss*, *msmo1*, *sqlea*, *hmgcra*, and *hmgcs1*—were upregulated in the lens of the *cryaba* ^−/−^; *nrf2* ^fh318/fh318^ line when compared to that of the WT (Figures 3B, C).

**FIGURE 4:**
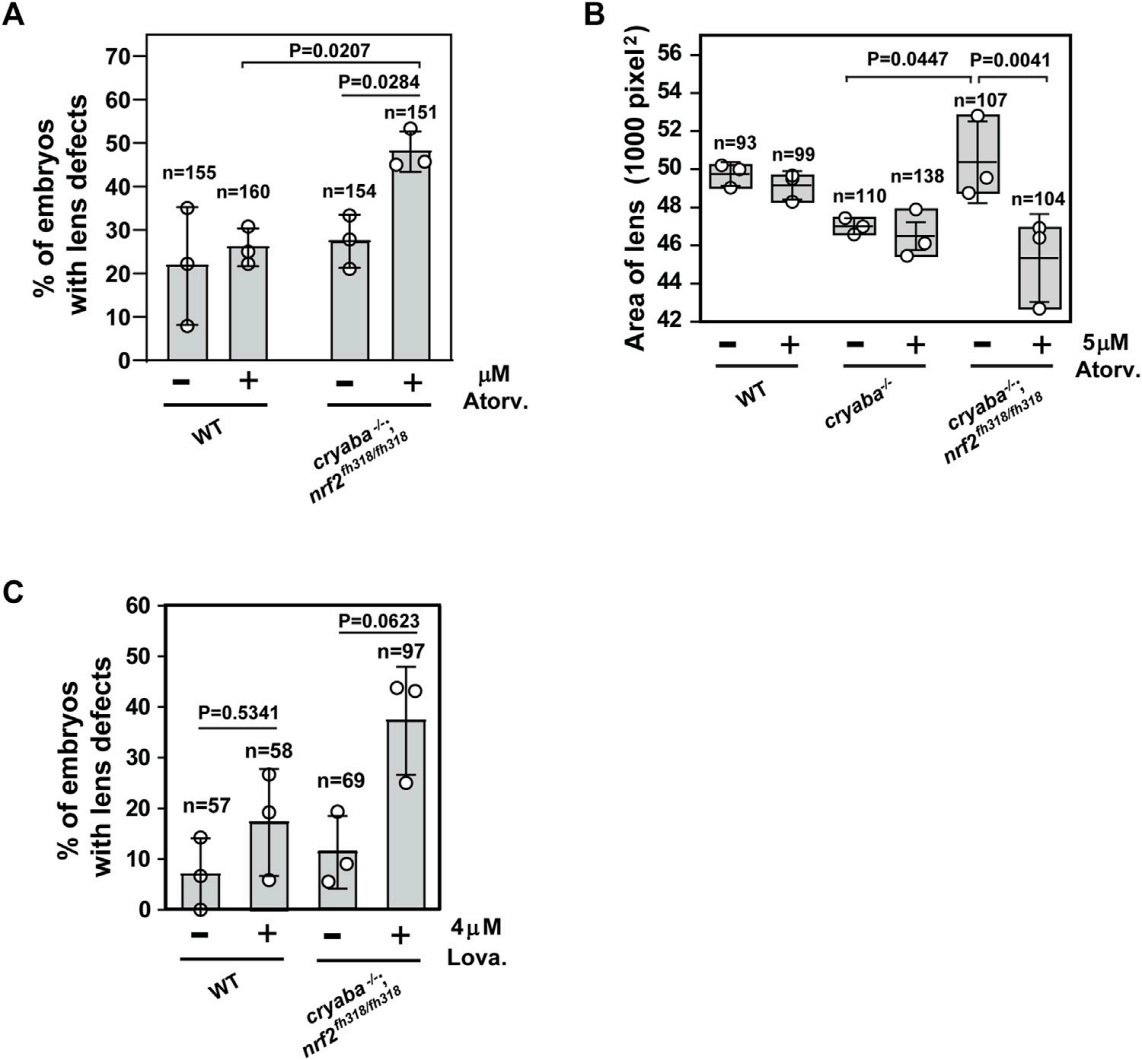
Increased penetrance of lens defects in *cryaba* ^−/−^; *nrf2* ^fh318/fh318^ in response to treatment of statins. **(A)** Zebrafish embryos were treated with either vehicle (1% DMSO) or 5 μM atorvastatin from 36 h post fertilization (hpf) until 4 dpf. Percentage of embryos showing lens defects between WT and *cryaba* ^−/−^; *nrf2* ^fh318/fh318^ were compared at 4 dpf. **(B)** Size of the lens of WT, *cryaba* ^−/−^, and *cryaba* ^−/−^; *nrf2* ^fh318/fh318^ embryos was measured in the presence and absence of 5 μM atorvastatin treatment. **(C)** WT and *cryaba* ^−/−^; *nrf2* ^fh318/fh318^ embryos were treated with 4 μM lovastatin for 16 h before examining lens abnormalities at 4 dpf. Data are expressed as mean ± SD from three independent experiments. n numbers indicate the total number of embryos across the independent experiments. Statistical significance was calculated using two-way ANOVA.

Based on the RNA-seq data, we hypothesized a correlation between activated cholesterol biosynthesis and the alleviated lens defects in the *cryaba* ^−/−^; *nrf2* ^fh318/fh318^ line. To test this hypothesis, we investigated whether the lens of *cryaba* ^−/−^; *nrf2* ^fh318/fh318^ embryos exhibits lower tolerance to treatment with statins, which are competitive inhibitors of the HMG-CoA-R enzyme, an early rate-limiting step in cholesterol synthesis (Istvan and Deisenhofer, 2001). For this purpose, the *cryaba* ^−/−^; *nrf2* ^fh318/fh318^ embryos were challenged with two different statins, atorvastatin and lovastatin, to inhibit the sterol biosynthetic pathway. *cryaba* ^−/−^; *nrf2* ^fh318/fh318^ embryos, incubated with 5 μM atorvastatin from 1–4 dpf, showed increased lens abnormalities (Figure 4A). By contrast, vehicle-treated *cryaba* ^−/−^; *nrf2* ^fh318/fh318^ (DMSO control) or WT embryos incubated with the same regimen of atorvastatin remained predominantly normal. In addition, we noticed that atorvastatin-exposed *cryaba* ^−/−^; *nrf2* ^fh318/fh318^ embryos have significantly reduced lens area than the vehicle-treated control (Figure 4B). Similarly, *cryaba* ^−/−^; *nrf2* ^fh318/fh318^ embryos displayed a higher percentage of lens defects than the WT in response to 4 μM lovastatin (Figure 4C). Together, these results suggest that upregulation of the cholesterol synthesis pathway in the lens is a plausible mechanism involved in the suppression of lens abnormalities in *cryaba* ^−/−^; *nrf2* ^fh318/fh318^.

### Dexamethasone-induced cardiac edema of Nrf2-deficient zebrafish is potentiated by αB-crystallin deficiency

**FIGURE 5:**
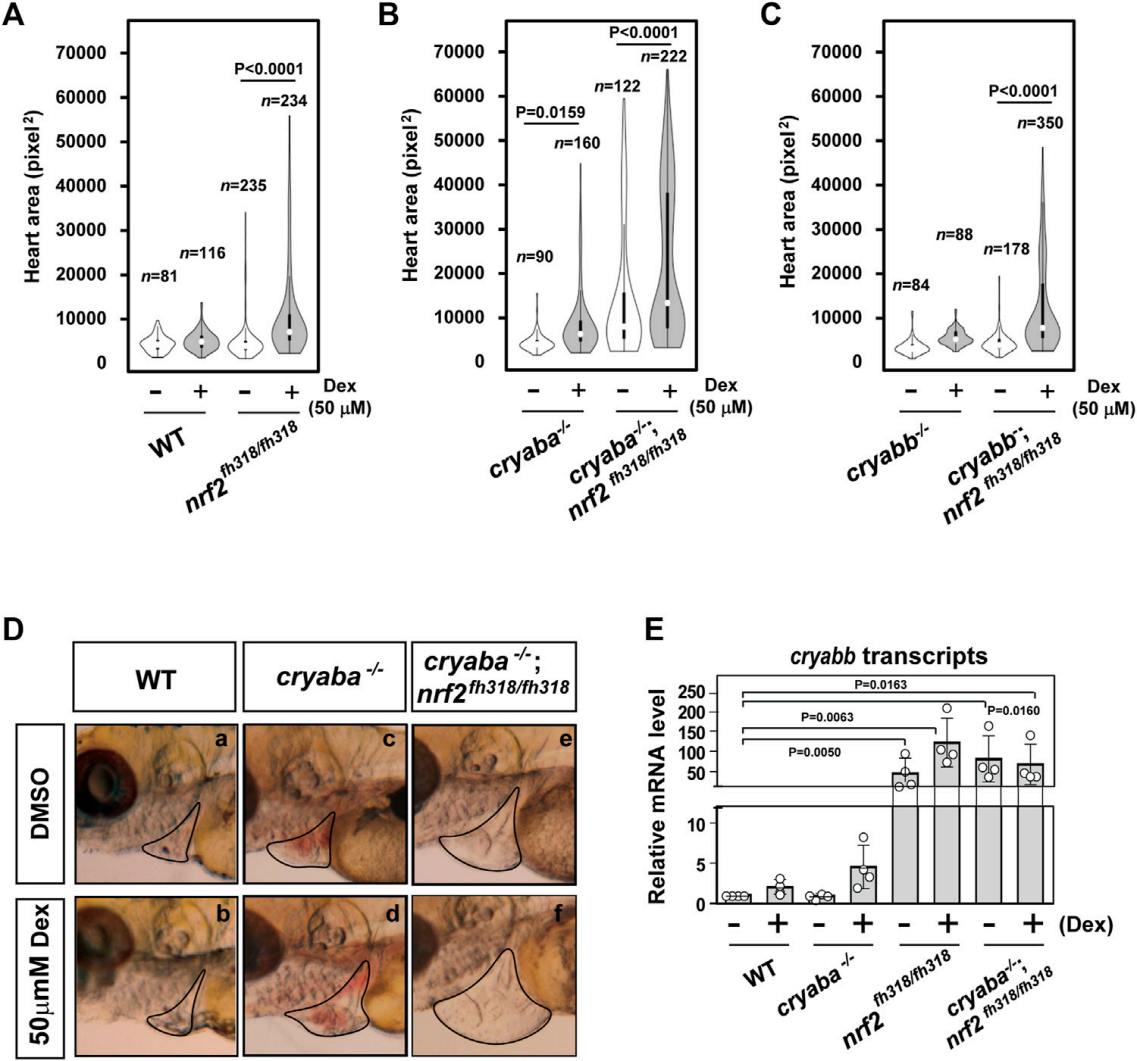
Nrf2 deficiency aggravates the heart edema phenotype of *cryaba* ^*−/−*^ fish in response to dexamethasone (Dex). The heart area of *nrf2* ^fh318/fh318^ embryos was measured following treatment with 50 μM Dex and compared to **(A)** the WT embryos, **(B)** *cryaba* ^−/−^ and *cryaba* ^−/−^; *nrf2* ^fh318/fh318^, and **(C)** *cryabb* ^−/−^ and *cryabb* ^−/−^; *nrf2* ^fh318/fh318^ following the same treatment regimen. **(D)** Representative images of cardiac phenotypes in WT, *cryaba* ^−/−^ and *cryaba* ^*−/−*^; *nrf2* ^fh318/fh318^ treated with either vehicle (DMSO) or Dex. **(E)** Relative changes in *cryabb* mRNA in 4 dpf embryos as measured by qRT-PCR. Data are expressed as mean ± SD obtained from three independent experiments. n numbers indicate the total number of embryos across the independent experiments. Statistical significance was calculated using two-way ANOVA.

In addition to lens defects, the loss of αB-crystallin function is associated with a cardiac phenotype that presents as embryonic cardiac edema (Mishra et al., 2018). The penetrance of this phenotype increases in response to stress induced by exposure to external glucocorticoid receptor agonists such as dexamethasone (Dex), as was previously described by Mishra et al. (2018). Because it has been reported that Nrf2 has a protective role in cardiac cells under oxidative stress (Chen and Maltagliati, 2018), we examined whether stress-induced heart edema of αB-crystallin KO lines is modulated by Nrf2 deficiency. For this purpose, we compared the heart areas (see the Methods section) of zebrafish embryos derived from the loss-of-function lines of αB-crystallins, Nrf2, and crosses of *cryaba* ^−/−^; *nrf2* ^fh318/fh318^ and *cryabb* ^−/−^; *nrf2* ^fh318/fh318^. All single KO lines and *nrf2* ^fh318/fh318^, treated with 50 μM dexamethasone (Dex) from 1–4 dpf, developed pericardial edema manifested by increased heart area relative to the WT embryos (Figures 5A–C). Remarkably, we observed a large increase in the penetrance of the phenotype in the *cryaba* ^−/−^; *nrf2* ^fh318/fh318^ line that was further accentuated in the presence of Dex (Figures 5B, D). Relative to *cryaba* ^−/−^; *nrf2* ^fh318/fh318^, cardiac edema was blunted in *cryabb* ^−/−^; *nrf2* ^fh318/fh318^ embryos treated with Dex. Furthermore, the vehicle-treated *cryabb* ^−/−^; *nrf2* ^fh318/fh318^ group retained a WT-like distribution (Figure 5C).

Because the discovery of *cryabb* mRNA upregulation in Nrf2-deficient heart tissues (Figure 1C) suggests that *cryabb* is transcriptionally activated in response to oxidative stress, we further explored the level of *cryabb* transcript in the presence of Dex. The transcription of *cryabb* mRNA appears to be upregulated with Dex treatment in WT (∼2-fold) and *cryaba* KO (∼3-fold) at 4 dpf (Figure 5E), although the *p*-values for the two-way ANOVA were higher than 0.05. Furthermore, *nrf2* ^fh318/fh318^ and *cryaba* ^−/−^; *nrf2* ^fh318/fh318^ embryos showed strongly upregulated *cryabb* mRNA regardless of the presence or absence of Dex (Figure 5E). Taken together, these results suggest that the two zebrafish αB-crystallin orthologues, *cryaba* and *cryabb*, have different regulatory mechanisms in the heart in response to oxidative stress. Our data suggest that *cryabb* functions as a stress–response gene regulated by both glucocorticoid stress and oxidative stress. Further detailed experiments are required to pinpoint the mechanism of the transcriptional control *cryabb* under different forms of stress.

### Changes in the extracellular region and tight junction pathways in the heart of cryaba^−/−^; nrf2^fh318/fh318^

**FIGURE 6:**
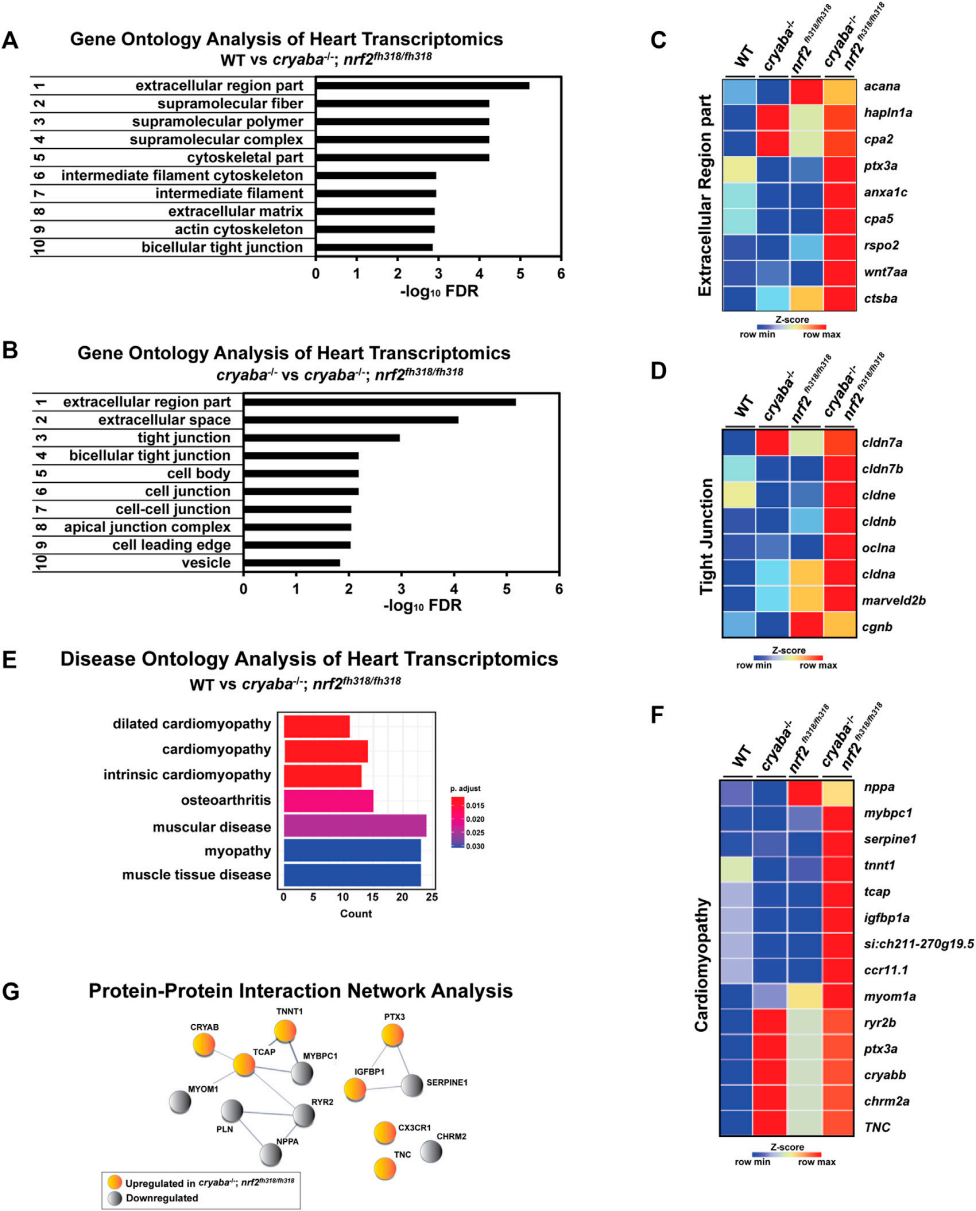
Transcriptome analysis reveals changes in genes belonging to the extracellular region in the heart of *cryaba* ^*−/−*^; *nrf2* ^fh318/fh318^. The top-ranked significant GO clusters in heart tissues between **(A)** WT versus *cryaba* ^−/−^; *nrf2* ^fh318/fh318^ and **(B)** *cryaba* ^−/−^ versus *cryaba* ^−/−^;*nrf2* ^fh318/fh318^. Heatmaps illustrate the transcripts in **(C)** the extracellular region GO term and **(D)** tight junction GO term. **(E)** Top terms in the Disease Ontology (DO) enrichment analysis highlight the number of genes enriched in each term (*x*-axis). The adjusted *p*-value of each term is indicated by color according to the legend. **(F)** The enriched genes in the cardiomyopathy DO cluster are illustrated by a heatmap. **(G)** Interaction between human homologs of transcripts in the cardiomyopathy DO term was assessed using STRING tool (Szklarczyk et al., 2021).

To gain mechanistic insight into the origin of the heart edema phenotype, we performed transcriptome analysis of heart tissues from adult zebrafish comparing WT, *cryaba* ^−/−^, *nrf2* ^fh318/fh318^, *and cryaba* ^−/−^; *nrf2* ^fh318/fh318^. RNA-seq analysis identified DE genes in the heart tissue of Nrf2-deficient zebrafish (*nrf2* ^fh318/fh318^ and *cryaba* ^−/−^; *nrf2* ^fh318/fh318^) that includes upregulation of *cryabb* transcripts, which is consistent with our data using the qRT-PCR analysis (Figure 1C, Supplementary Figure S8F). To derive a global understanding on how these genes affect heart health, the DE genes with a false discovery rate (FDR) cut off ≤ 0.05 between groups were used for further pathway analysis. The most significant gene ontology (GO) was calculated by using the WEB-based GEne SeT AnaLysis Toolkit (WebGestalt) (Wang et al., 2013). The top important GO terms between *cryaba* ^−/−^; *nrf2* ^fh318/fh318^ and WT included the extracellular region, supermolecular fiber, and bicellular tight junction (Figure 6A). The extracellular region and tight junction pathways were also listed in the most enriched GO cluster between *cryaba* ^−/−^; *nrf2* ^fh318/fh318^ versus *cryaba* ^−/−^ (Figure 6B). These findings suggest a significant alteration of genes related to extracellular interactions in the heart of *cryaba* ^−/−^; *nrf2* ^fh318/fh318^ embryos.

To identify the extracellular components which are exclusively changed in the heart tissue of *cryaba* ^−/−^; *nrf2* ^fh318/fh318^, the subset of genes in the GO term within the extracellular region from each comparison (*cryaba* ^−/−^; *nrf2* ^fh318/fh318^ vs. WT, and *cryaba* ^−/−^; *nrf2* ^fh318/fh318^ vs. *cryaba* ^−/−^) was sorted by a Venn-diagram analysis (Supplementary Figure S9; Supplementary Tables S3, S4). The result identified *cpa5*, *cstba*, *cpa2*, *anxa1c*, *hapln1a*, *ptx3a*, *rspo2*, *wnt7aa*, and *acana* genes, which are closely involved in the degradation, remodeling, or inflammation of the extracellular matrix (ECM) (Figure 6C). In addition, the transcripts which encode components in the tight junction, such as *cldn7b*, *cldn3*, *cldnb*, *oclna*, *cagnb*, and *marveld2b*, showed higher expressions in the heart tissues of *cryaba* ^−/−^; *nrf2* ^fh318/fh318^ than *cryaba* ^−/−^ (Figure 6D; Supplementary Figure S10). These results suggest that molecular changes of the ECM and tight junction pathways in the heart in *cryaba* ^−/−^; *nrf2* ^fh318/fh318^ correlate with greater penetrance of the cardiac edema phenotype.

Finally, we performed Disease Ontology (DO) analysis with known disease markers to explore the potential pathological mechanism underlying enhanced heart edema in *cryaba* ^−/−^; *nrf2* ^fh318/fh318^. For this purpose, the DE genes between *cryaba* ^−/−^; *nrf2* ^fh318/fh318^ and WT in the heart tissues were analyzed using DOSE (Yu et al., 2015) to calculate the enrichment of DO terms. The results confirmed that the transcriptional changes in the heart of *cryaba* ^−/−^; *nrf2* ^fh318/fh318^ are significantly associated with muscle diseases, specifically cardiomyopathy (Figures 6E, F). We also noticed that the human homologs of transcripts in the cardiomyopathy DO are functionally clustered via STRING, a database of protein–protein interactions (Szklarczyk et al., 2021) (Figure 6G). This result further supports the transcriptional changes in *cryaba* ^−/−^; *nrf2* ^fh318/fh318^ being functionally associated with heart health.

## Discussion

### Zebrafish *αB-crystallin* genes are regulated under oxidative stress in a tissue-specific manner

Despite their ubiquitous expression, the physiological roles of sHSPs beyond chaperone activity continue to be enigmatic. While they have been implicated in numerous stress responses, there has been a lack of systematic investigation of how they are coupled to cellular protective pathways. To address this outstanding question, we have utilized zebrafish as a tractable model system to dissect the role of αB-crystallin both in the lens and other tissues. In a previous work, we demonstrated that αB-crystallins are critical for lens development and that the loss of function of αB-crystallins compromises the resistance of the heart to stress (Mishra et al., 2018; Zou et al., 2015). Here, we explored the role of the two αB-crystallins in response to oxidative stress signaling. The finding of tissue-specific transcriptional link between *nrf2* and *cryabb* but not *cryaba* in zebrafish is novel and consistent with the recent findings that Nrf2 can function as a repressor (Brown et al., 2008; Liu et al., 2018). Equally novel is the identification of molecular pathways modulated in loss-of-function zebrafish lines. The results presented here suggest that *cryabb* plays a role in the stress response in the brain and heart tissues, but *cryaba* mainly has a function limited to the lens in zebrafish, most likely as a chaperone. Overall, our results set the stage for further studies on how *nrf2* and *cryabb* are coupled in the zebrafish lens and heart. In both tissues, long-lived cells experience sustained oxidative loads that lead to a number of pathologies.

Human αB-crystallin is found in the mitochondria of both the lens and retinal cells (McGreal et al., 2012). Specifically, αB-crystallin interacts with cytochrome c both *in vitro* and *in vivo*, and its overexpression helps maintain mitochondrial membrane potential during oxidative stress (McGreal et al., 2012). Thus, exploring mitochondria changes in zebrafish αB-crystallin loss of function under stress of Nrf2 deficiency or other oxidative-challenged conditions will be critical.

### A novel intersection between lens integrity and cholesterol biosynthesis pathway

A major finding of this work is that lens defects in the *cryaba* ^*−/−*^ line were rescued in the *cryaba* ^−/−^; *nrf2* ^fh318/fh318^ line (Figure 2). Furthermore, RNA-seq analysis correlated this rescue with the upregulation of the cholesterol biosynthesis pathway (Figure 3). Following up on the hypothesis of a connection between cholesterol biosynthesis and alleviated lens defects in *cryaba* ^−/−^; *nrf2* ^fh318/fh318^, we utilized statins and HMG-CoA reductase inhibitors to lower cholesterol synthesis. We determined that treatment with atorvastatin or lovastatin increased the penetrance of lens defect in *cryaba* ^−/−^; *nrf2* ^fh318/fh318^.

The beneficial role of high cholesterol in the lens has been described and specifically linked to a reduction in oxygen transport across membranes to protect against oxidative damage (Dotson et al., 2017; Widomska et al., 2017; Widomska and Subczynski, 2019). Moreover, elevating cholesterol precursors, particularly lanosterol, has been studied as a means to reverse protein aggregation in cataract (Widomska et al., 2017; Kang et al., 2018; Widomska and Subczynski, 2019; Miyashita et al., 2022). In this context, a genetic mutation in lanosterol synthase (*lss*) is associated with cholesterol deficiency–associated cataracts (Mori et al., 2006). However, the precise molecular mechanism linking cholesterol biosynthesis and cataract remains largely unknown. Here, we provide an additional connection between cholesterol and lens integrity using high-throughput transcriptome profiling. Given that lens RNA-seq analysis revealed upregulation of cholesterol biosynthetic enzymes, we propose that lathosterol is likely the upregulated sterol in the lens, although the means by which Nrf2 deficiency upregulates cholesterol biosynthetic enzymes is still unclear. Thus, further experiments are required to delineate the intersection between the cholesterol synthesis pathway and Nrf2 in the lens.

Our results are consistent with previous reports that have uncovered Nrf2-dependent regulation of cholesterol biosynthesis in mouse liver and in cultured liver cells. A pharmacogenomics investigation of the Nrf2 activator 3H-1,2-dithiole-3-thione (D3T) in mice showed RNA enrichment of enzymes to be involved in cholesterol biosynthesis, whereas no effect on these enzymes was observed as a consequence of treatment with the Nrf2 activator 1-[2-cyano-3,12-dioxooleana-1,9 (11)-dien-28-oyl]-imidazole (CDDO-Im) (Wible et al., 2018). By contrast, a dietary supplement found to activate Nrf2 downregulated the cholesterol biosynthesis pathway (Hybertson et al., 2022). The contrasting results notwithstanding, these studies confirm the relationship between Nrf2 and the cholesterol biosynthesis pathway and point to the need for a more thorough understanding of how Nrf2 regulates this pathway.

Zebrafish is a popular model for studies on embryonic development, brain function, and disease progression. This is due to its many advantages, such as rapid development, easy-to-manipulate genetics, suitability for live imaging, and chemical screening. However, there are limitations in analyzing tissue-specific transcriptomics or proteomics at the embryonic stage. Even with the use of single-cell RNA sequencing technology, it is difficult to detect low-expressed transcripts. In this study, we conducted screening of embryo phenotypes, such as lens defects and heart edema. We also performed tissue-specific transcriptome analyses on the lens or heart tissues from adult zebrafish. However, phenotype screening was carried out on embryos, and pathway analysis was conducted in adult tissues. Thus, further experiments, such as histological analysis of lens or heart tissues from adult *cryaba* ^−/−^ zebrafish, are required to better understand the molecular and cellular pathways affected as a result of αB-crystallin loss of function in an oxidative stress context of *nrf2* deficiency.

### Loss of Nrf2 function increases the penetrance of the cardiac phenotype in *cryaba* ^*−/−*^ but not *cryabb* ^*−/−*^ zebrafish

In contrast to the lens phenotype, the fraction of embryos displaying stress-induced heart edema in *cryaba* ^*−/−*^ was increased by the loss of Nrf2 function. More importantly, a higher penetrance of the heart defect phenotype was observed in *cryaba* ^−/−^; *nrf2* ^fh318/fh318^ embryos, even without Dex treatment (Figure 5B). Furthermore, the search for disease-associated molecular pathways revealed cardiomyopathy as the top DO cluster in *cryaba* ^−/−^; *nrf2* ^fh318/fh318^ adult zebrafish heart, suggesting a detrimental synergetic effect of the depletion of *nrf2* and *cryaba* in heart tissue.

While more studies are required to mechanistically understand the link between these various pathways, our findings can be included in the extensive literature that implicates αB-crystallin in the oxidative balance of cardiomyocytes in mouse lines as well as in humans (Rajasekaran et al., 2007; Dimauro et al., 2018; Yin et al., 2019). First identified in investigations of the human cardiomyopathy mutant R120G of αB-crystallin (Fardeau et al., 1978; Vicart et al., 1998), a link between αB-crystallin and reductive stress has been elaborated in subsequent studies (Rajasekaran et al., 2007; Bauersachs and Widder, 2010; Xie et al., 2013; Morrow et al., 2015). Zebrafish is an ideal model to investigate this link further.

## Materials and methods

### Zebrafish maintenance and breeding

AB wild-type strain zebrafish (*Danio rerio*) were used. The embryos were obtained by natural spawning and raised at 28.5°C on a 14:10 h light/dark cycle in egg water 30 mg/L instant ocean in deionized water containing 0.003% PTU (w/v) to prevent pigment formation. Embryos were staged according to their ages (in dpf). The following mutant and transgenic fish lines were used: *cryaba* ^vu612^ (*cryαba* ^−/−^); *cryabb* ^vu613^ (*cryαbb* ^−/−^); *nrf2* ^fh318^. All animal procedures were approved by the Vanderbilt University Institutional Animal Care and Use Committee.

### Quantitative reverse-transcription PCR

Zebrafish were euthanized, and the lens, heart, and brain tissues were dissected as described by Gupta and Mullins (2010). Tissues were immediately snap-frozen in liquid nitrogen, and RNA was extracted from all samples simultaneously using TRIzol (Invitrogen) and RNA Clean & Concentrator Kit (Zymo Research). A total of 500 μg of total RNA was then used as a template with the SuperScript III First-Strand Synthesis kit (Invitrogen) to produce cDNA. The specific targets were amplified by RT-PCR using oligonucleotides as given in Supplementary Tables S1, S2. *β-actin* was used as the internal control. The ANOVA and t-tests were performed to calculate the *p*-value to determine the significant difference between samples.

### RNA-seq

Total RNA from zebrafish tissues was isolated simultaneously using TRIzol (Invitrogen) and RNA Clean & Concentrator kit (Zymo Research). RNA-Seq libraries (*n* = 2) were processed at the Vanderbilt Technologies for Advanced Genomics (VANTAGE) core. Briefly, the samples were processed using the TruSeq Standard Sample Prep Kit (Illumina) to prepare cDNA libraries after Poly(A) selection. The libraries were sequenced on an Illumina NovaSeq 6000 to a depth of 50 million at 150 bp paired-end reads per library. For lens RNA-seq, FASTQ reads were aligned through Illumina’s DRAGEN RNA Seq pipeline. EdgeR (3.30.3) packages were used to measure differential gene expression with genes that achieved a count per million mapped reads (CPM). For heart RNA-seq, Vanderbilt Technologies for Advanced Genomics Analysis and Research Design (VANGARD) performed the DEG and further analyses.

### Gene and Disease Ontology

For lens RNA seq, DEG (FDR cutoff ≤ 0.01) was analyzed through the use of IPA (QIAGEN, https://www.qiagenbioinformatics.com/products/ingenuitypathway-analysis). The GO clusters with the significant *p-*value were taken for further analysis. To use the human Disease Ontology database, the zebrafish genes were first matched to their human orthologues using Ensembl BioMart (www.ensembl.org/biomart). Then, the gene set of human orthologues was processed using Bioconductor DOSE packages (Yu et al., 2015) with a *p*-value cutoff of ≤ 0.05 to identify the corresponding DO terms.

### Microscopy and image processing

Lenses of live embryos in 0.3× Danieau water with PTU/tricaine were analyzed by using bright field microscopy (Zeiss Axio Zoom V16) at 4 dpf. The percent of lens defects was scored as defined in our previous study (Mishra et al., 2018). Briefly, the phenotypic characteristic manifested as a spherical, shiny droplet spreading across the lens, which was classified as a defect. For heart imaging, the heart area of the live embryos at 4 dpf was analyzed by using bright field Axio Zoom microscopy. Quantification of the size of the heart area was performed by ImageJ (Duan et al., 2016; Incardona and Scholz, 2016).

### Drug treatments

One dpf embryo was manually dechorionated, and 10 embryos were placed in one well of a 24-well plate (polystyrene, tissue culture grade) with 1 mL of 0.3× Danieau water. Then, 50 μM dexamethasone (Sigma, D1756) diluted in 0.3× Danieau water was treated from 1 to 4 dpf to examine the cardiac phenotypes. For stain experiments, 2.5 or 5 μM atorvastatin (Santa Cruz Biotechnology, sc-337542A) was treated from 1 to 4 dpf to observe lens defects, and 4 μM lovastatin (Santa Cruz Biotechnology, sc-200850A) was treated for 16 h before examining lens abnormalities at 4 dpf.

### Statistics

Statistical analyses were carried out with GraphPad Prism software 9 (GraphPad) by means of Student’s *t*-test or ANOVA. Comparison between groups was performed with Bonferroni or Tukey test for one-way or two-way ANOVA. Statistical significance was defined as *p* < 0.05.

## Data Availability

RNA-seq data have been deposited in the ArrayExpress database at EMBL-EBI (www.ebi.ac.uk/arrayexpress) under accession number E-MTAB-12172. Processed RNA-seq data of lens and heart tissues are provided as Supplementary Tables S5, S6.

## Abbreviations

sHSPs, small heat shock proteins; dpf, days post fertilization; qRT, quantitative reverse transcription; PTU, 1-phenyl-2-thiourea; Dex, dexamethasone; tBHP, tert-Butyl hydroperoxide; GO, Gene Ontology; RNA-seq, RNA-sequencing; Atorv, atorvastatin; Lova, lovastatin; FDR, false discovery rate.
